# A study of differential circRNA and lncRNA expressions in COVID-19-infected peripheral blood

**DOI:** 10.1038/s41598-021-86134-0

**Published:** 2021-04-12

**Authors:** Yingping Wu, Tiejun Zhao, Riqiang Deng, Xiaoping Xia, Bin Li, Xunzhang Wang

**Affiliations:** 1 College of Medicine, The Fourth Affiliated Hospital Zhejiang University School of Medicine, Zhejiang University, Hangzhou, China; 2 School of Life Science, Sun Yat-sen University, Guangzhou, 510275 China

**Keywords:** Biomarkers, Diseases, Clinical genetics, Gene expression, Genetic interaction, Sequencing

## Abstract

To conquer the worldwide outbreak of COVID-19 virus, a large number of studies have been carried out on COVID-19 infection, transmission and treatment. However, few studies have been conducted from the perspectives of circRNA and lncRNA, which are known to be involved in regulating many life activities, such as immune tolerance and immune escapes, and hence may provide invaluable information in the emerging COVID-19 infection and recurrence. Moreover, exosomes has been reported to play an important role in COVID-19 recurrence, and thus may interact with the expression of circRNA and lncRNA. In this work, we sequenced circRNA, lncRNA and mRNA from recurrent COVID-19 patients and healthy people, and compared the differences. GO and KEGG enrichment analysis show that differentially expressed circRNA and lncRNA are mainly involved in the regulation of host cell cycle, apoptosis, immune inflammation, signaling pathway and other processes. The comparison to exosomes related databases shows that there are 114 differentially expressed circRNA, and 10 differentially expressed lncRNA related to exosomes. These studies provide reference for exploring circRNA and lncRNA to study the infection mechanism of COVID-19, their diagnostic and therapeutic values, as well as the possibility to employ them as biomarkers.

## Introduction

Since December 2019, the Novel Coronavirus, abbreviated as COVID-19, has spread across the globe. It poses a serious threat to the public health of all countries in the world, which has attracted high attention from both governments and academic communities. More and more researchers are joining, together with continuously increasing investment from various resources, in the battle against this virus, in order to suppress its impact on the human health/life and worldwide economy to the largest extent.

Firstly, researchers have been popularizing the basic knowledge of coronavirus, and introducing the characteristics of coronavirus, the diseases caused by coronavirus, and COVID-19 infection clinical diagnosis, source tracing, characteristics and development trend of infection in different regions^1–11^.

Secondly, researchers have started to sequence the genome of COVID-19, based on which the genome structure and protein functionalities of COVID-19 can be analyzed in detail. For example, Bin, et al. performed such studies by means of bioinformatics^12^. Baruah, et al. took an immunoinformatics approach to identify significant cytotoxic T lymphocytes (CTL) and B-cell epitopes in the COVID-19 surface glycoprotein^13^. Paraskevis, et al. carried out the genomic evolution analysis^14^. Djomkam, et al. pointed out that angiotensin converting enzyme 2 (ACE2) and transmembrane protease serines 2 (TMPRSS2) played an important role in the process of COVID-2019 entering the cell^15^. Dong, et al. analyzed the origin and pathogenicity of COVID-19 from the aspects of genome and protein structure model^16^. Li, et al. studied the expression of COVID-19 receptor ACE2 in fetal organs through a single-cell transcriptome^17^. Ghafouri-Fard, et al. reviewed the ACE2 and COVID-19 infection, and clarified in details the important role of ACE2 and recent research progress in this arena^18^. Noroozi, et al. pointed out that immune response differences have been found at the levels of several cytokines between severely affected COVID-19 patients and those with moderate or mild symptoms^19^. Wang, et al. pointed out that two potential drugs that can inhibit COVID-19 could be filtered out by in vitro experiment of the Wuhan Institute of Virology. The first is the broad-spectrum anti-RNA viral drug, remdesivir. The second is chloroquine phosphate, an anti-malarial drug^20^. Wu, et al. compared the genetic differences between COVID-19 and SARS by sequencing the genes^21^. Li, et al. analyzed the differential expression of microRNA in the peripheral blood of COVID-19 patients^22^.

The last, which is also the most important, is the study of COVID-19 medical treatment. Vaidya, et al. reported the successful treatment of severe COVID-19 infection with Clazakizumab in a heart transplant patient^23^. Zhao, et al. pointed out that Lymphocyte antigen 6 complex,locus E,LY6E (LY6E) is a key effector of antiviral immunity. ArfGAP Domain-Containing Protein 2 (ADAP2), gamma-interferon-inducible lysosomal thiol reductase (GILT), and LY6E are three cellular proteins known to be able to interfere with virus activity. Experiments have shown that overexpression of LY6E in HEK 293 cells can inhibit the entry of HCoV-OC43 into host cells, while overexpression of GILT or ADAP2 does not^24^. Mantlo, et al. found in their study that the type I interferon pathway plays an important role in inhibiting SARS-CoV-2 infection^25^. Based on the knowledge accumulated from previous research, Liu, et al. proposed a possible emergency prevention and treatment regimen for severe acute respiratory infection caused by COVID-19^26^. Zhao, et al. described the treatment of COVID-19 patients with corticosteroids^27^. Zumla, et al., reported that host-directed therapies should be an option in reducing COVID-19 mortality^28^. Gurwitz, et al. introduced angiotensin receptor blockers in the treatment of diseases caused by COVID-19^29^. In addition, the treatment using remdesivir, favipiravir and other drugs was also the focus of the analysis by researchers^30,31^.

Although extensive research efforts have been invested all over the world, and some vaccines have been developed, it remains far away from the target of a full understanding of the COVID-19 virus. A lot more efforts are required to explore each important aspect. Especially, the recently observed world-wide recurrence of COVID-19 patients presents a severe challenge to the proper treatment and urgent needs to thoroughly understand and eliminate COVID-19. To a large degree, the recurrence of COVID-19 reflects the immune interactions between the virus and the host, including immune escape etc. CircRNA is a type of circular non-coding RNA formed by covalently binding of 3′ and 5′ ends after reverse splicing^32^. LncRNAs are non-coding RNAs with lengths greater than 200 nucleotides^33^. Note that circRNA and lncRNA have been found to be able to regulate immune activities such as immune tolerance and immune escape, etc.^34,35^. Thus, a careful study on COVID-19 from the aspects of circRNA and lncRNA can reflect the immune state before and post infection, and is essential to identify those molecules that dominate the regulation of those important life activities. So far, there has not been any detailed study focused on the differential expression of circRNA and lncRNA in recurrent COVID-19 patients. This study will analyze the difference between circRNA and lncRNA, and its evolution in the human body after the person is infected, so as to provide a new perspective for a comprehensive understanding of the infection mechanism of COVID-19, as well as to provide new ideas for seeking treatment solutions and judging the effects of treatment. On the other hand, it has been reported that exosomes^36^ can behave as a diagnosis marker for COVID-19. Thus, we also investigated exosomes related differential expressions of circRNA and lncRNA in this study, serving as a reference for the management and control of COVID-19 infection and recurrence.

## Materials and methods

In this study, total RNA was extracted from the whole blood of three recurrent COVID-19 patients and three healthy people for further microarray analysis. The whole blood (2 mL) was collected from all donors by using BD PAXgene blood RNA tubes (BD, cat. no. 762165).

The Department of Clinical Laboratory is a China National Accreditation Service (CNAS) Conformity Assessment -certified laboratory, and the ISO 15,189 system has been in stable operation in this laboratory over 3 years. All medical staff involved in PCR testing were authorized and qualified professionals obtained official permission to conduct these tests in China. Written informed consent was obtained from all patients, and the study protocol was approved by the Ethics Committee of Zhejiang University (Approval No. K20200026), as attached in supplementary file 1. The experimental methods were conducted in accordance with approved guidelines.

### Sequencing and differential analysis methods

Total RNA was isolated using RNeasy Total RNA Isolation Kit (Qiagen, GmbH, Germany)/ TRIzol reagent (Life Technologies, Carlsbad, CA, U.S.), and purified using a RNeasy Mini Kit (Qiagen, GmbH, Germany), following the manufacturer’s instructions. Total RNA was checked for a RIN (RNA Integrity Number) to inspect RNA integration by an Agilent Bioanalyzer 2100 (Agilent Technologies, Santa Clara, CA, U.S.). RNA samples from each group were then used to generate biotinylated cRNA targets for the Sino Human ceRNA array V3.0. The biotinylated cRNA targets were then hybridized with slides. After hybridization, slides were scanned on the Agilent Microarray Scanner (Agilent Technologies, Santa Clara, CA, U.S.). Data were extracted with Feature Extraction software 10.7 (Agilent Technologies, Santa Clara, CA, U.S.). Raw data were normalized by quantile algorithm, R package “limma”. The microarray experiments were performed by following the protocol of Agilent Technologies, Inc. at Sinotech Genomics Corporation^37,38^.

Genes with a fold change of at least 2 were selected for further analysis. Heatmap plots were obtained by a R package “pheatmap” for the target genes.

Gene Ontology (GO)\ pathway enrichment analysis were done using Fisher's precision test by a R package “clusterProfiler” for the target genes. GO categories \Pathway with Fisher's precision test with *p* values < 0.05 were selected.

In the sequencing analysis, primers for circRNA were designed according to circBase, while that for lncRNA were designed based on NONCODE, Ensemble, NCBI, and LNCipedia.

### GO and KEGG functional enrichment analysis methods

We performed a GO analysis for biological processes, cellular components and molecular function and a KEGG pathway analysis (Kyoto Encyclopedia of Genes and Genomes http://www.genome.ad.jp/kegg) via enrich R package.

### Co-expression analysis method

A co-expression network diagram is used to calculate the co-expression relationship between genes according to the dynamic changes of gene expression signal values, and get the expression regulation relationship and direction between genes, so as to build the expression regulation network of genes. Using the co-expression network diagram, the researchers could obtain the core regulatory genes of the samples that change with the experiment, by analyzing the gene regulation ability. Specifically, Pearson correlation coefficient can be calculated based on the expressions of mRNA and lncRNA/circRNA, and the first 400 relationship pairs with a correlation greater than 0.9 and a *p* value less than 0.05 were selected for a detailed map using Cytoscape.

### ceRNA analysis method

A computational mechanism was established to use circRNA/lncRNA as ceRNA to protect mRNA from microRNA degradation. Based on the expression value of genes, the regulation network of microRNA sponge adsorption was established through regression model analysis and seed sequence matching. Different types of ceRNA mechanisms between the experimental group and the control group were studied to find specific ceRNA. Specifically, the expression value is used to establish the relationship among circRNA/lncRNA, mRNA, and microRNA, which is then used to infer whether microRNA regulates circRNA/lncRNA and mRNA, and find out the relationship between the three.

Seed sequence matching analysis: microRNA and mRNA target gene relationship, microRNA and circRNA target gene relationship.

miRNADA software was employed to predict the differential lncRNA/circRNA targeted miRNA, and select the first 400 relationship pairs with binding energy ranging from small to large, and made a detailed map with Cytoscape.

### Ethics approval and consent to participate

The study design was approved by Ethics Committee of Zhejiang University. Written informed consent was obtained from all patients. We have read and understood your journal’s policies, and we believe that neither the manuscript nor the study violates any of these. This manuscript has not been published or presented elsewhere in part or in entirety and is not under consideration by another journal.

## Results

### Differential gene

**Figure 1:**
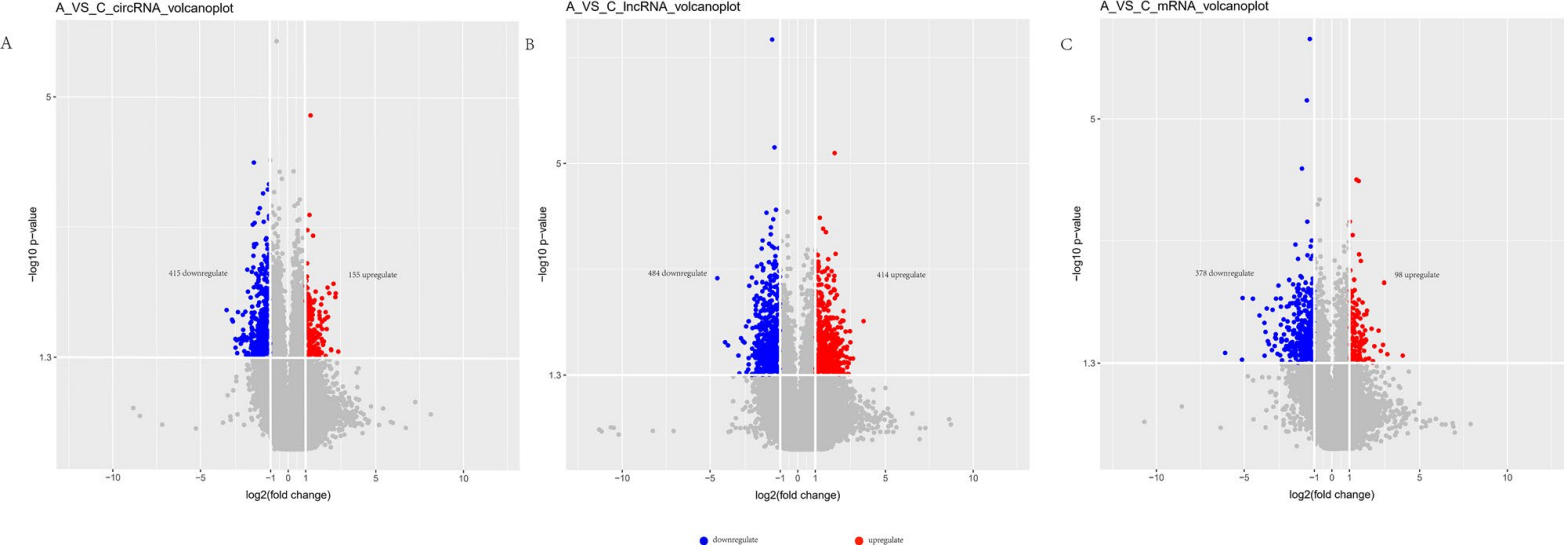
Volcano plots of different RNA types. (**A**) circRNA; (**B**) lncRNA; (**C**) mRNA; down- and up- regulations are marked with blue and red colors, respectively. Fold change (linear) ≥ 2, *p* value < 0.05. Samples are from 3 recurrent COVID-19 patients and 3 healthy people.

After normalization, statistical methods such as fold-change (expression difference multiple) and T-test (Student's T-test) were used for screening and statistical significance statistics of the normalized data. The selection criteria were as follows:

1. Fold Change (linear) is less than or equal to 0.5 or greater than or equal to 2, indicating that the differentially expressed genes are up-regulated or down-regulated, respectively, by more than 2 times.
2. P-value of T-test is less than 0.05 or 0.01, generally taking 0.05 as the standard. Through comparison and analysis of the positive and healthy groups in accordance with the above standard, as shown in Fig. 1, we found a total of 570 circRNA that have difference, among which 155 are up-regulated and 415 are down-regulated; and 898 lncRNA that have difference, in which 414 are up-regulated and 484 are down-regulated; and 476 mRNA that have difference, in which 98 are up-regulated and 378 are down-regulated.

Note that we did not observe clear difference in ACE gene between the experimental group and the control group, as expected, because the study was performed on the whole blood from recurrent patient instead of cells from particular tissues of patients who got infected or re-infected.

### GO and KEGG functional enrichment

In the GO and KEGG processes, the enrichment analysis was performed on the function of the parent gene of circRNA, while on the function of lncRNA aided by its target gene, and on the function of the gene of mRNA, respectively.

**Figure 2:**
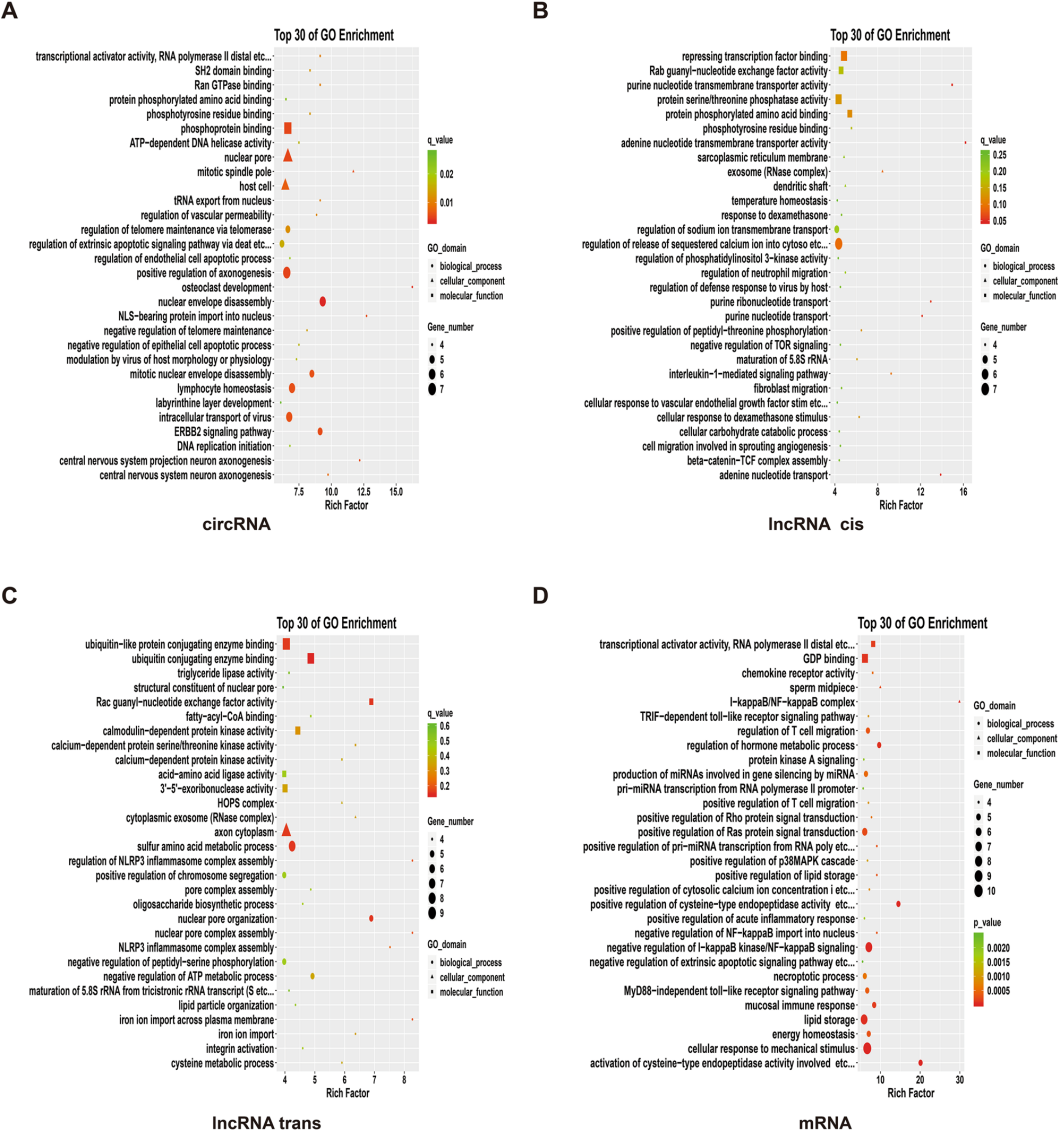
GO enrichment result. (**A**) circRNA; (**B**) lncRNA cis; (**D**) lncRNA trans; (**D**) mRNA. The employed methodology is Fisher precision verification. Data package is clusterProfiler, from R (version 3.4.3); the selection standards include that the differential gene count dropped on certain term is not less than 2, and *p* value is smaller than 0.05. The term in the drawing is arranged in descending order of size according to the value of an enrich factor, and takes the first 30 results. Samples are from 3 recurrent COVID-19 patients and 3 healthy people.

As shown in Fig. 2, the main differentially expressed circRNA functions ranked among top 30 in the GO enrichment analysis are as follows. The first aspect is its impact on signaling pathways, such as the central nervous system neurons and the formation of the projection neurons axon, and the ERBB2 signaling pathway, and the function of SH2 domain and so on, which further affect the signal transmission. The second aspect is the impact on protein formation and processing, such as phosphorylation, the transport of tRNA from the nucleus output, RNA polymerase II and other effects on protein formation and processing. The third aspect is the influence on DNA replication, such as the initiation of DNA replication, ATP-dependent DNA helicase activity and so on, and thus the influence on DNA replication. The fourth aspect is to influence the growth, apoptosis and morphology of host cells, such as the disintegration of mitotic nucleus, nuclear envelope disassembly, endothelial cell and epithelial cell apoptosis, as well as regulation of extrinsic apoptotic signaling pathway via Deat etc. In addition, it will affect 1) the embryonic development, by affecting the development of the labyrinthine layer; 2) the telomeres by affecting regulation of telomere maintenance via telomerase and negative regulation of telomere maintenance; 3) NLS-bearing protein import into nucleus, etc.; 4) the transport of virus in cells, lymphocyte homeostasis, osteoclast development; and 5) the modulation of vascular permeability, modulation by virus of host morphology or physiology, etc.

The functional enrichment of lncRNA indicates that it had an effect on signal transduction in cis-regulation, such as Wnt/ -catenin signaling pathway, Ras signaling pathway, interleukin-1-mediated signaling pathway, PI3K/AKT/mTOR signaling pathway and TOR signaling pathway. It will affect 1) the transport of substances, such as the transport of purine nucleotides, the transport and release of sodium and calcium ions; 2) the phosphorylation of proteins, such as phosphorylation of serine and threonine; 3) the migration of cells, such as fibroblasts, neutrophils, etc.; 4) the formation of proteins, such as the effects in repressing transcription factor binding, and maturation of 5.8 S rRNA, etc., thus affecting the protein transcription of translation. It also affects the production of blood vessels, such as cellular response to vascular growth factor stimulation, etc. In addition, it will affect exosomes, host's defense response to the virus, and sugar metabolism.

In terms of trans-regulation, differentially expressed lncRNA functions affect the metabolism of substances, such as 1) the effects on triglyceride lipase activity and thus on fat metabolism; 2) the ubiquitin-conjugating enzyme binding and ubiquitin-like protein conjugating enzyme binding, thus affecting protein metabolism; 3) interfering with oligosaccharide biosynthetic process, thus further affecting sugar metabolism; 4) the effect on ribonuclease, which further influences the degradation of RNA; 5) affecting the synthesis of protein, such as affecting the activity of amino acid ligase and maturation of 5.8 S rRNA from tricistronic rRNA transcript, which further impacts on the formation of protein; 6) influence on signal transduction, such as effect on integral protein, calcium-dependent protein kinase and Rac guanyl-nucleotide exchange factor activity, etc., thereby affecting signal transduction; 7) influence on nuclear pore and chromosome separation; and 8) impact on the transport of iron ions. In addition, it affects negative regulation of ATP metabolic process, and hence affecting energy metabolism. Its enrichment on homotypic fusion and protein sorting (HOPS) complex, implies changes on the autophagy process of cells. Additionally, its impact on the NOD-like receptor protein 3 (NLRP3) inflammasome complex assembly affects the host's natural immunity.

The functions of mRNA that produced difference change are mainly concentrated in the following aspects. The first aspect is the metabolism of substance and energy, such as the differentiation in GDP binding, showing that there are differences in energy metabolism, and the differentiation in the aspects of regulation of the body process, fat storage, positive regulation of cysteine-type endopeptidase activity, etc., and regulation of the body process, showing that substance metabolism is affected. The second aspect is related to signal transduction, mainly in the MyD88-independent toll-like receptor signaling pathway, I-κB kinase/NF-κB signaling pathway, p38MAPK signaling pathway, Ras signaling pathway, Rho signaling pathway, TRIF-dependent toll-like receptor signaling pathway, and protein kinase A signaling. The third aspect is related to immune, such as mucosal immune response and regulation of T cell migration, etc. The fourth aspect is related to the production of miRNAs, such as positive regulation of pri-miRNA transcription from RNA poly, etc., production of miRNAs involved in gene silencing by miRNAs, etc. Moreover, it is related to transcription, which is reflected in the relatively high functional enrichment in transcriptional activator activity, RNA polymerase II distal, etc. The concentration of calcium ions also play a role, since the enrichment is higher positive in the regulation of cytosolic calcium ion concentration, etc. It is also related to the hydrolysis of the peptide chain due to the high enrichment on the activation of cysteine-type endopeptidase activity involved. It is worth noting that there are also higher differences in enrichment in the middle section of sperms and chemokines.

**Figure 3:**
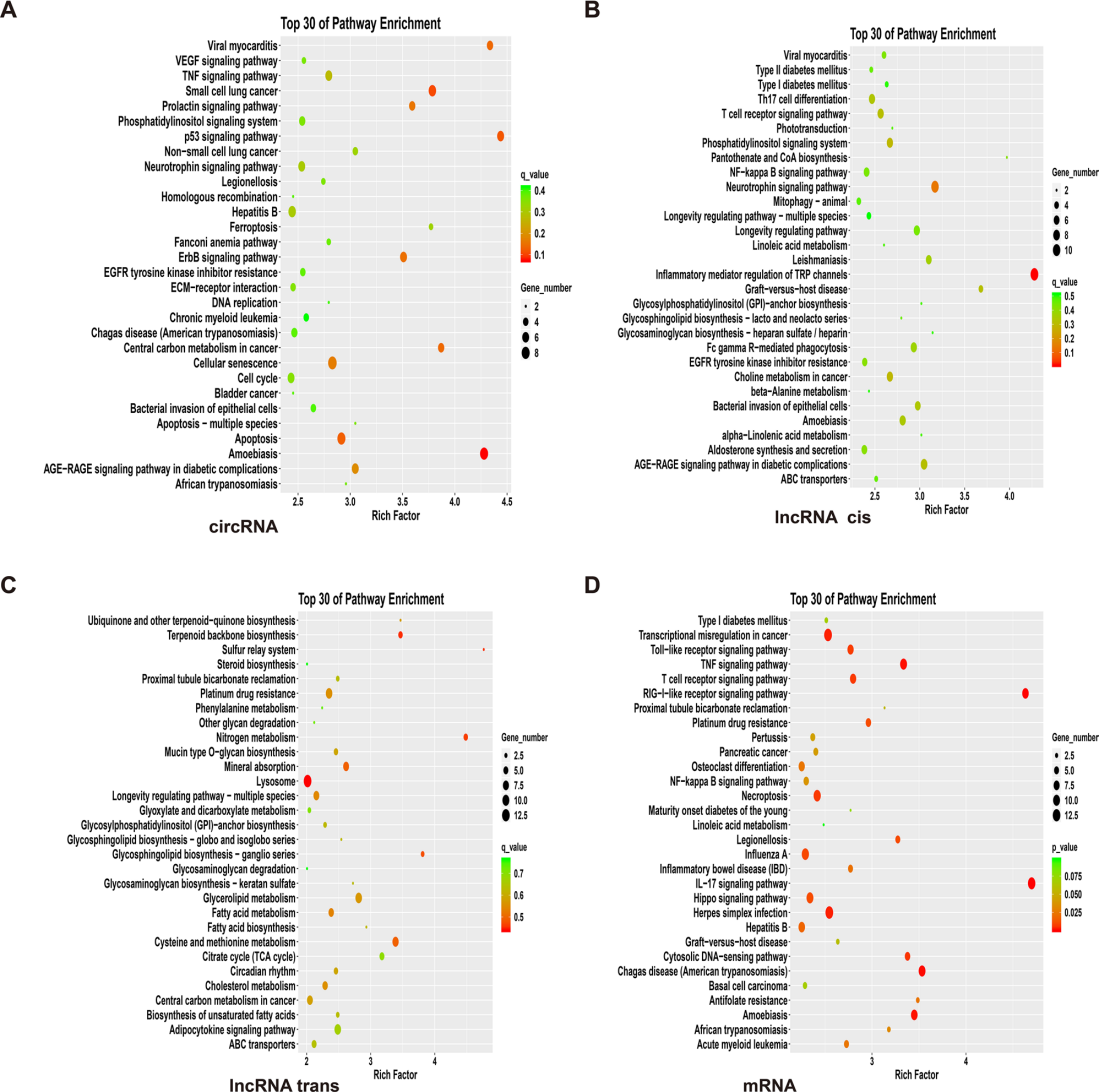
KEGG enrichment result. (**A**) circRNA; (**B**) lncRNA cis; (**C**) lncRNA trans; (**D**) mRNA. The selection standards include that the differential gene count dropped on certain term is not less than 2, and *p* value is smaller than 0.05. The term in the drawing is arranged in descending order of size according to the value of an enrich factor, and takes the first 30 results. Samples are from 3 recurrent COVID-19 patients and 3 healthy people. Data package is clusterProfiler, from R (version 3.4.3).

As shown in Fig. 3, in the KEGG enrichment analysis, differentially expressed circRNA functions ranked in the top 30, mainly include the following aspects. One aspect is associated with a multitude of diseases, such as African trypanosomiasis (sleeping sickness), amoebiasis (dysentery), Chagas disease (American Trypanosomiasis), chronic myeloid leukemia, Fanconi anemia, hepatitis B, cancer, small cell lung cancer, and viral myocarditis. Among these diseases, malignant tumors and diseases caused by parasites, viruses and bacteria are the main outcomes. The second aspect is related to cell death, apoptosis, cycle and senescence, such as ferroptosis, which is a tosis of cell death caused by iron-dependent oxidative damage, different from apoptosis, necrosis, and autophagy. The third aspect has to do with the number of signaling pathways, mainly including the AGE-RAGE signaling pathway in diabetic activity, ErbB signaling pathway, p53 signaling pathway, TNF signaling pathway, VEGF signaling pathway, phosphatylinositol signaling system, and prolactin signaling pathway and so on. In addition, there were also higher enrichment in DNA replication, homologous recombination, ECM-receptor interaction, neurotrophin signaling pathway, EGFR tyrosine kinase inhibitor resistance, etc.

The KEGG enrichment of lncRNA, in terms of cis-regulation, shows it is related to diseases such as amoebiasis, graft-versus-host disease, leishmaniasis, viral meningitis, type I and type II diabetes—mainly diseases and immune disorders caused by parasites and viruses. The second aspect is also related to various signaling pathways, such as longevity regulating pathways, NF-κB signaling pathway, T cell receptor signaling pathway, phototransduction, and Th17 cell differentiation, among others. The third aspect is related to the metabolic synthesis of substances, such as pantothenate and CoA biosynthesis, glycosaminoglycan biosynthesis-heparan sulfate, heparin, linoleic acid metabolism, beta-alanine metabolism, etc. In addition, there is also a higher enrichment in terms of ABC transporters, Fc gamma R-mediated phagocytosis, mitophagy-animal, etc. The KEGG enrichment of lncRNA has overlap with that of circRNA in the aspect of the AGE-RAGE signaling pathway in the diabetic activity, bacterial invasion of epithelial cells, and neurotrophin signaling pathway.

In terms of trans-regulation, it is mainly related to the anabolism of substances, such as the Krebs cycle (TCA cycle), fatty acid metabolism, ubiquinone and other terpenoid-quinone biosynthesis, etc. It can be seen that major macromolecules and related metabolic pathways are involved in the organism. In addition, lysosomes, platinum drug resistance, and the circadian rhythm are also highly enriched. It has overlaps with the cis-regulation of lncRNA in the aspect of ABC transporters, etc., and with the KEGG enrichment of circRNA, in terms of central carbon metabolism in cancer, etc.

The KEGG enrichment of differentially expressed mRNA is mainly concentrated in the following aspects. On the one hand, it is related to disease, such as amoebiasis and pancreatic cancer, which intersects with (such as hepatitis B and legionellosis,) and differs from (such as basal cell carcinoma, herpes simplex infection and Influenza A.) those covered by circRNA and lncRNA. The second aspect is the signaling pathway, such as the NF-κB signaling pathway, IL-17 signaling pathway, toll-like receptor signaling pathway, and T cell receptor signaling pathway, etc., which also intersects with (such as NF-κB signaling pathway and TNF signaling pathway) and differs (such as RIG-I-like receptor signaling pathway) from the circRNA- and lncRNA-containing diseases. In addition, there is also a high enrichment in osteoclast differentiation, cytosolic DNA-sensing pathway, etc.

**Figure 4:**
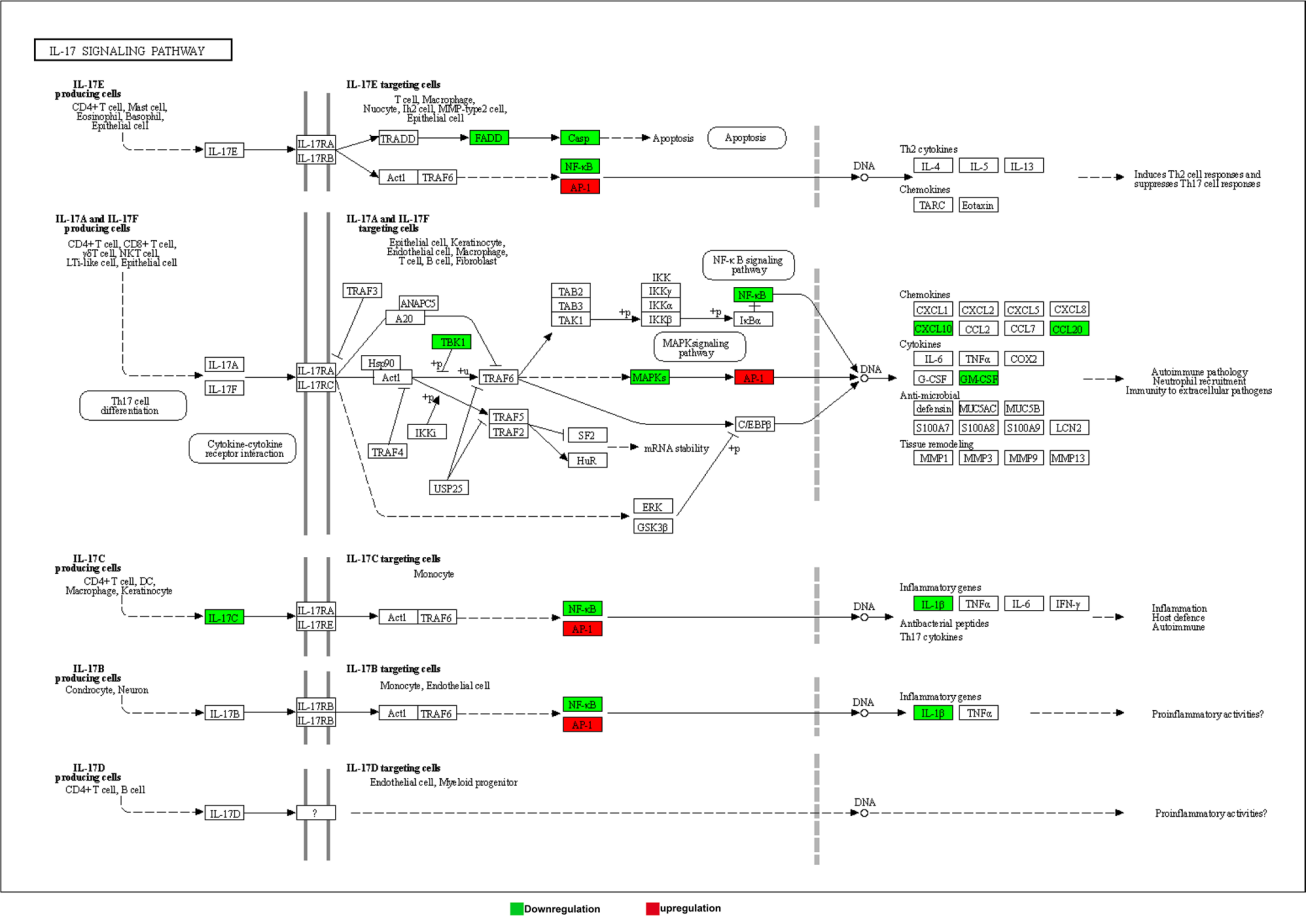
IL-17 signaling pathway. Down- and up-regulations are marked with green and red colors, respectively. Samples are from 3 recurrent COVID-19 patients and 3 healthy people.

To sum up, after the patient is infected by COVID-19 virus, some circRNA and lncRNA will be affected, thus playing a role in multiple immune and inflammatory pathways, the growth and apoptosis of host cells, and negatively impacting the normal physiological activities of the host. For example, immune and inflammatory pathways including toll-like receptor signal transduction, T cell receptor signal transduction, NF-κB signal transduction, and inflammasome signal transduction are affected, leading to adverse effects on host autoimmunity. And in these immune and inflammatory pathways, some more important genes are involved, such as Interleukin-1 (IL1) and NF-κB, which are related to multiple pathways. IL1, also known as lymphocyte stimulator, is a class of cytokines produced by and used by a variety of cells, mainly in the form of IL-1α and IL-1β. Its primary roles include the following aspects: 1) It provides immune regulation at low local concentration, through the means of synergistic stimulation of the activation of APC and T cells, promoting B cell proliferation and secretion of antibodies. However, a large amount of production of IL1 can produce endocrine effects including inducing acute phase protein synthesis in the liver, causing fever and cachexia^39,40^. NF-κB (enhanced κ-light chain of nuclear factor-activated B cells) is a protein complex that controls transcriptional DNA, cytokine production and cell survival. NF- κB is present in almost all animal cell types and is involved in cellular responses to stimuli such as stress, cytokines, free radicals, heavy metals, ultraviolet radiation, oxidized LDL, and bacterial or viral antigens. NF-κB plays a key role in regulating the immune response to infection. Incorrect regulation of NF- κB is associated with cancer, inflammatory and autoimmune diseases, septic shock, viral infection and immune dysplasia. NF-κB is also associated with synaptic plasticity and memory processes^41,42^. As shown in Fig. 4, down-regulation of IL-17C affects NF-κB as well as IL1β in the IL-17 signaling pathway. NF-κB is also down-regulated affected by other genes, which further influences downstream gene expression, and hence the host's autoimmunity^43,44^. It suggests that this is one of the approaches that is worthy of further study.

Based on the above, we learned the changes of circRNA, lncRNA and mRNA of the patient after infected with COVID-19 virus. The mRNA changes are very likely due to the effects of circRNA and lncRNA. One of the main ways for circRNA to play its role is to regulate target genes with the help of miRNA, while lncRNA also plays its role with the help of miRNA. In addition, it can bind to target genes to achieve the regulation of target genes. For this, we carried out prediction on the differentially expressed circRNA and lncRNA binding miRNA, and the cis/trans target genes of differentially expressed lncRNA. By means of co-expression and ceRNA, the relationship between them was studied.

### Co-expression of analysis

By using the co-expression analysis method, we further analyzed the co-expression relationship between circRNA, lncRNA and mRNA, and understood the expression regulation relationship and direction between genes, so as to construct the gene expression regulation network. The co-expression network diagram can be used to analyze the gene regulation ability to obtain the core regulatory genes of the samples that change with the experiment.

**Figure 5:**
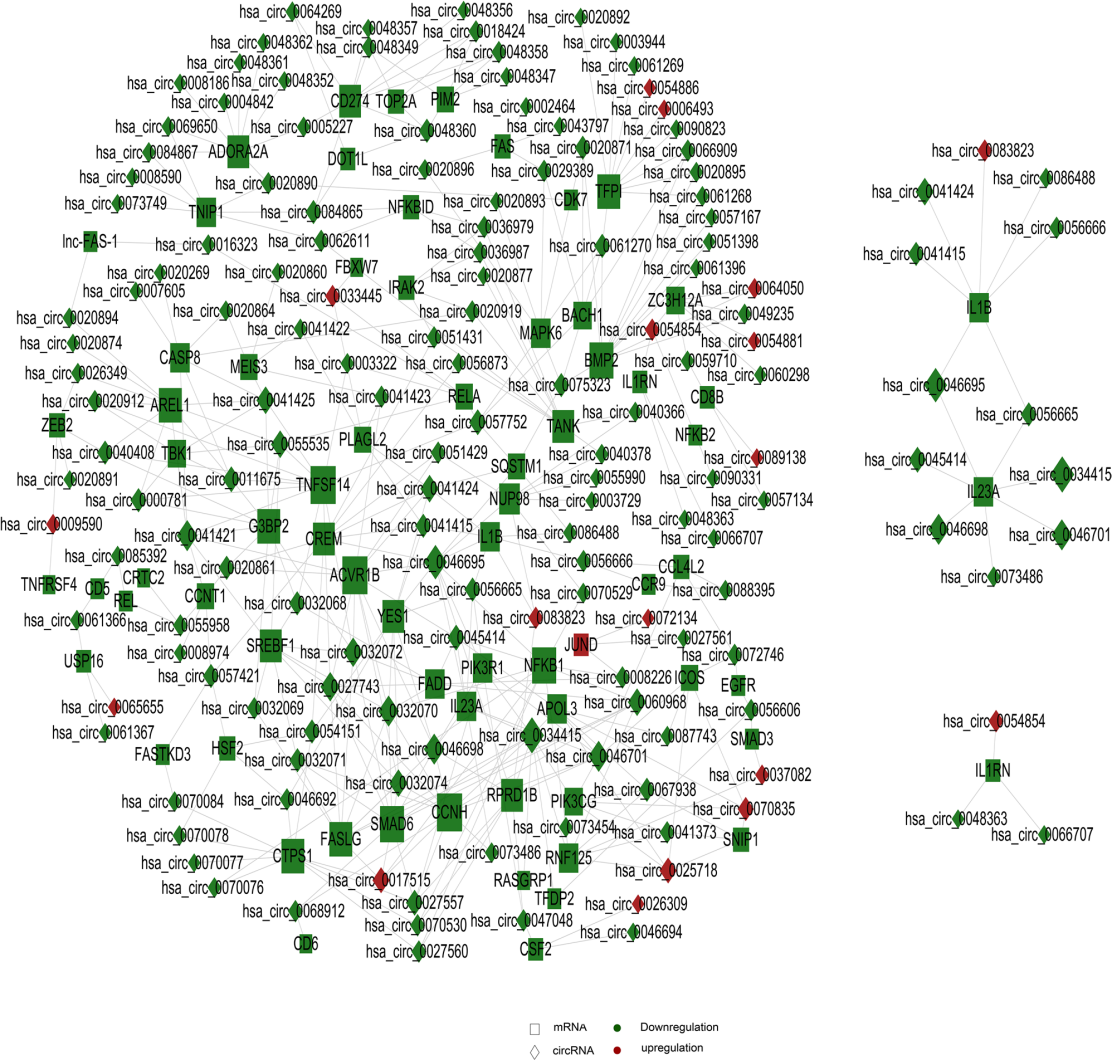
circRNA co-expression network diagram. Rectangle represents circRNA, square represents mRNA; Red represents up-regulation and green represents down-regulation. Each dot in the network represents a gene, and the size of the circle represents the gene's ability to interact with other genes. We use degree to quantify it, and the larger the degree, the more genes interact with other genes. Based on the degree of each gene in the gene synergy network, key genes in the network can be obtained. Samples are from 3 recurrent COVID-19 patients and 3 healthy people.

On the basis of GO and KEGG analysis, we first selected GO_term and pathway related to cell cycle and immune inflammation as the objects for further analysis, to study the co-expression relationship between circRNA and lncRNA and mRNA. Specifically, we selected correlation pairs with a correlation greater than 0.9 and a *p* value less than 0.05. For those with relation pairs more than 400, the first 400 pairs are selected to draw the diagram. If relation pairs are less than 400 pairs, then select all. Cytoscape was chosen for the fine drawing. From the relation between circRNA and mRNA shown in Fig. 5, it can be observed that genes such as NF-κB, IL1β, Phosphatidylinositide 3-kinases (PI3K), and Tumor Necrosis Factor Ligand Superfamily, Member 14 (TNFSF14) have received relatively significant impact.

**Figure 6:**
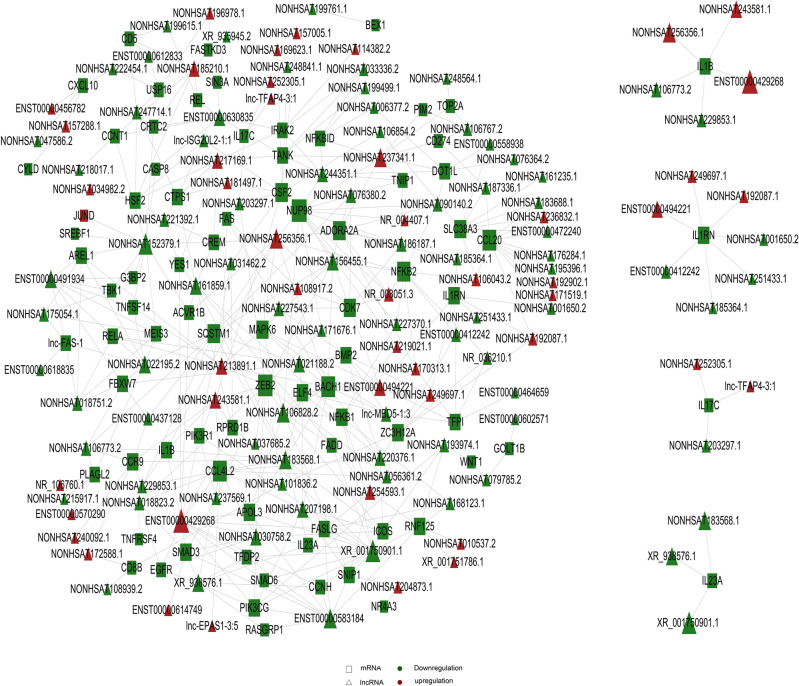
lncRNA co-expression network diagram. Triangle represents lncRNA, square represents mRNA; red represents up-regulation and green represents down-regulation. Each dot in the network represents a gene, and the size of the circle represents the gene's ability to interact with other genes. We use degree to quantify it, and the larger the degree, the more genes interact with other genes. Based on the degree of each gene in the gene synergy network, key genes in the network can be obtained. Samples are from 3 recurrent COVID-19 patients and 3 healthy people.

As shown in Fig. 6, in the correlation between lncRNA and mRNA, NF-κB, IL1β, PI3K, and TNFSF14 and other genes are also included.

### ceRNA analysis

**Figure 7:**
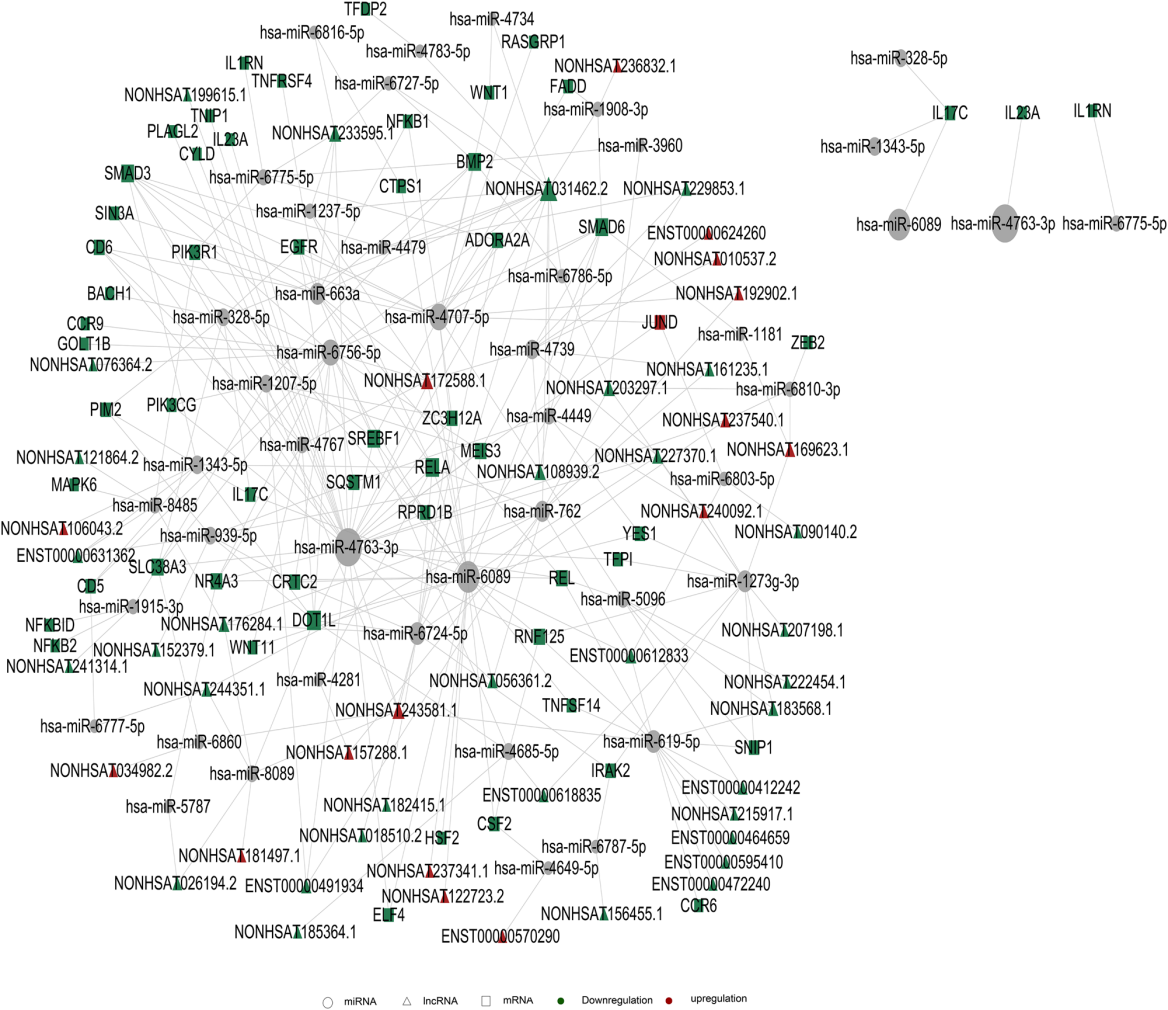
ceRNA analysis results of lncRNA. Triangle represents lncRNA, square represents mRNA, circle represents miRNA; red represents up-regulation and green represents down-regulation. Each dot in the network represents a gene, the lines represent microRNA that are regulated between genes. The size of the circle represents the gene's ability to interact with other genes. We use degree to quantify it, and the larger the degree, the more genes interact with other genes. Based on the degree of each gene in the gene synergy network, key genes in the network can be obtained. Samples are from 3 recurrent COVID-19 patients and 3 healthy people.

With the help of ceRNA analysis, the relationship among lncRNA, mRNA, and microRNA was established to infer whether lncRNA regulates microRNA and ultimately influences mRNA. All the relation pairs of these three are found out and ranked according to the binding energy following the small-to-large order. For those with relation pairs more than 400, the first 400 pairs are selected to draw the diagram. If relation pairs are less than 400 pairs, then select all. Again, Cytoscape is used to make fine drawings. As shown in Fig. 7, on the basis of GO and KEGG analysis, we first selected GO_term and pathway related to cell cycle and immune inflammation as the objects for further analysis, and further demonstrated the relationship among them with ceRNA analysis. In this figure, we can see that many differentially expressed genes are changed through the regulation of lncRNA on miRNA. For example, IL1RN is regulated by has-Mir-6775-5p, while has-Mir-6775-5p is regulated by differentially changed PIK3R1, and eventually regulated by the differentially changed lncRNA, such as ENST00000631362. NF-kB is regulated by miRNA such as has-miR-4707-5p, and finally regulated by the differentially changed lncRNA such as NONHSAT122723.2.

**Figure 8:**
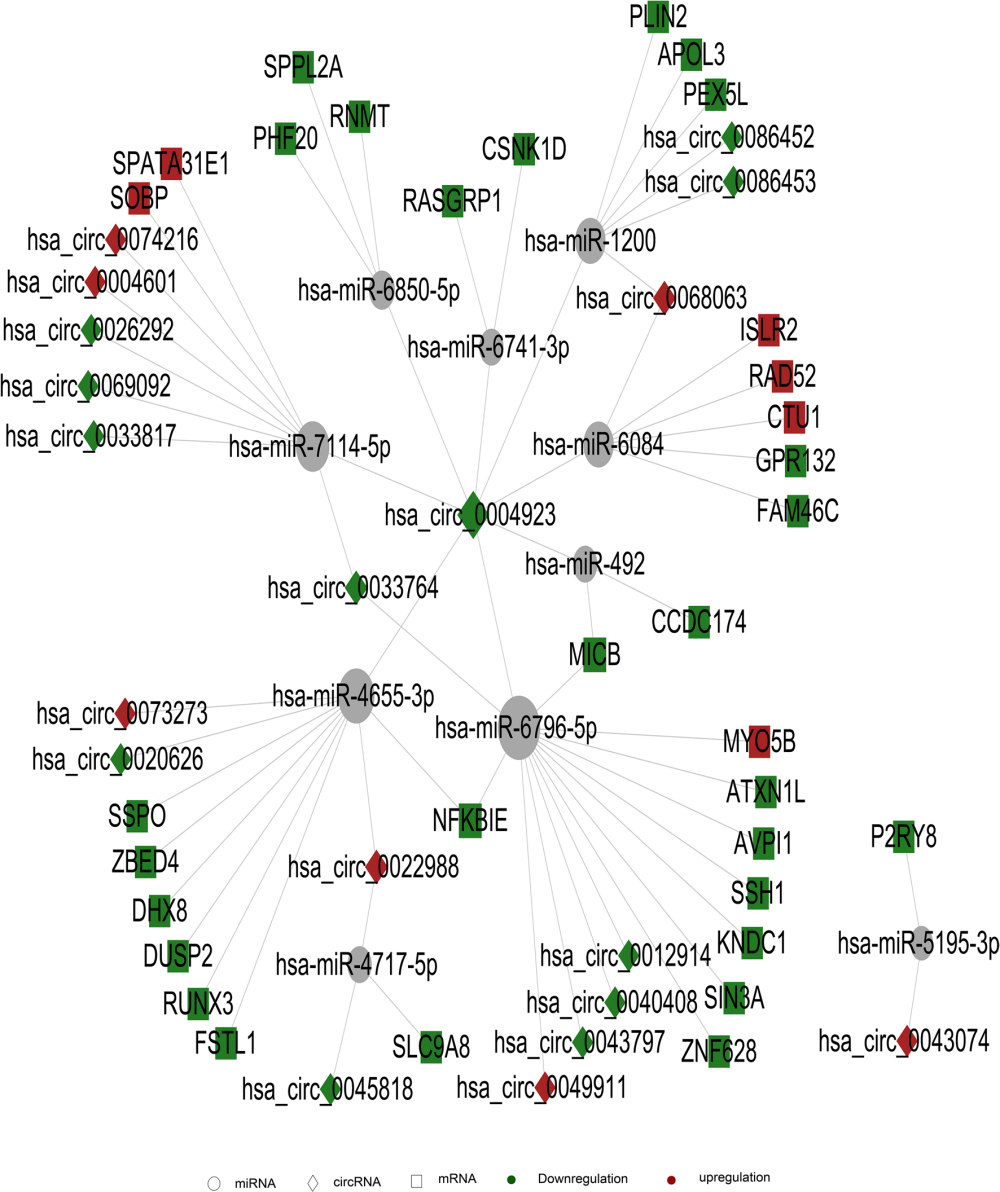
ceRNA analysis results of circRNA. Rectangle represents circRNA, square represents mRNA, circle represents miRNA; red represents up-regulation and green represents down-regulation. Each dot in the network represents a gene, the lines represent microRNA that are regulated between genes. The size of the circle represents the gene's ability to interact with other genes. We use degree to quantify it, and the larger the degree, the more genes interact with other genes. Based on the degree of each gene in the gene synergy network, key genes in the network can be obtained. Samples are from 3 recurrent COVID-19 patients and 3 healthy people.

We also used ceRNA analysis to establish the relationship among circRNA, mRNA, and microRNA, so as to infer whether circRNA regulates microRNA and ultimately affects mRNA. A similar procedure was performed in relation pair selection plot drawing, as has been detailed above. As shown in Fig. 8, on the basis of GO and KEGG analysis, we first selected GO_term and pathway related to cell cycle and immune inflammation as the objects for further analysis, and further demonstrated the relationship among them with ceRNA analysis. In this figure, we can see that many differentially expressed genes are changed through circRNA regulation of miRNA. For example, has-Circ-0004923 can act on has-Mir-6796-5p, thereby affecting the expression of many genes including NFKBIE. However, in relation pairs, we did not find the regulation of NF-κB and IL1-related genes required for screening. This may also be related to the current amount of known circRNA in the circRNA database, and it cannot be excluded that circRNA in the possible location may exert regulatory effects on these genes, or affect those genes in some other way.

### The relationship with exosomes

Through the enrichment analysis above, we observe that differentially expressed circRNA and lncRNA are closely linked with diseases such as cancer. Studying these diseases provides us important help to understand the pathogenesis of COVID-19. In addition, exosomes are found to be playing a role in the result of lncRNA cis GO enrichment.

Exosomes refer to small membrane vesicles (30–150 nm) containing complex RNA and proteins, which, nowadays, specifically refers to disc-like vesicles with diameters of 40–100 nm. The function of exosomes depends on the cell type from which they originate, and they can be involved in immune response, antigen presentation, cell migration, cell differentiation, tumor invasion and other aspects. Studies have shown that exosomes from tumor sources participate in the exchange of genetic information between tumor cells and basal cells, leading to the formation of a large number of new blood vessels and promoting the growth and invasion of tumors^45,46^. In addition, some researchers also pointed out that exosomes play a very important role in the process of viral infection. On the one hand, exosomes can pass the virus nucleic acid and protein, and may change the microenvironment, promoting the spread of infection. On the other hand, exosomes can cause body's immune response through the activation of antiviral or the transfer of antiviral molecules^47,48^. After COVID-19 infected a person, circRNA and lncRNA related to exosomes were also expressed in a different way. This could provide reference for understanding viral infection and collective immune process, and provide a direction for further research on diagnosis, treatment and prevention of diseases. Some researchers pointed out that exosomes could be used as markers of disease diagnosis. In other words, some specific blood drawing indicators in the blood could be used to judge the improvement of the disease^49,50^. This may also be the case with regard to COVID-19 infection. It has been reported that exosomes could be a treatment option based on their study of COVID-19 and exosomes^51,52^. Moreover, some researchers observed the difference in exosomes before and after infection, and pointed out that exosomes could be an important factor for the relapse^53^. Based on this, we compared the differentially expressed circRNA and lncRNA with the relevant databases (http://www.exorbase.org/) and ExoCarta (http://www.exocarta.org/) to find circRNA and lncRNA related to exosomes.

In further analysis, we used the exoRBase (http://www.exorbase.org/) and ExoCarta (http://www.exocarta.org/) database to annotate lncRNA and circRNA found in exosomes that had been studied to provide reference for subsequent studies, particularly on exosomes. We classified 114 differentially expressed cirRNA and 10 differentially expressed lncRNA related to exosomes. For example, hsa_circ_0001313 plays a role in promoting the growth and metastasis of gastric cancer, colon cancer, etc., and can be used as a promising biomarker for the determination of treatment status and infection with the virus^54,55^.

## Discussion

In this study, we sequenced the blood of patients infected with COVID-19 virus and of healthy people. By sequencing, we analyzed the differences of circRNA, lncRNA and mRNA between the patients and healthy people, and further analyzed the relationships between circRNA and mRNA, the relation among circRNA, mRNA and miRNA, the relation between lncRNA and mRNA, and the relation among LncRNA, mRNA and miRNA. However, since miRNA was not sequenced separately, the difference and change of miRNA could not be demonstrated, which could be analyzed in further studies. In addition, known databases were chosen in our comparison, and the differentially expressed circRNA and lncRNA have been reported. In other words, unknown circRNA and lncRNA have not been predicted. In the diseases caused by the COVID-19 virus, there may be some new circRNA and lncRNA that play a regulatory role, which can be analyzed in a further study.

Regarding COVID-19 infection of host cells, numerous researchers have carried out extensive studies on the mechanism. Many previous studies of other viruses have shown that the expression of cell genes changes rapidly during latent viral infection. However, from the aspects of circRNA and lncRNA, there has not been much involvement. In this study, through comparison, we found differentially expressed circRNA and lncRNA, indicating that these two RNAs may play an important role in this mechanism.

CircRNA and lncRNA have been shown to be cross-species. Changes in circRNA’s expression have been reported in a number of diseases, including cancer, heart disease and neurological disorders, although the underlying mechanisms have not been disclosed. It has also been reported in some cases of viral infection. For example, Lou, et al. found that circRNA is differentially expressed in THP-1 cells during HCMV incubation and in the weak infection condition. By KEGG analysis of differentially expressed circRNA host genes, researchers identified 18 pathway circRNA host genes that were enriched among 300 differentially expressed genes. In this study, by careful comparison, we found 570 circRNA with differences, among which 155 are up-regulated, 415 are down-regulated; 898 differentially expressed lncRNA, among which 414 are up-regulated, 484 are down-regulated; and 476 differentially expressed mRNA, among which 98 are up-regulated and 378 are down-regulated. With the aid of the GO and KEGG, we further performed functional enrichment on these differentially expressed circRNA, lncRNA, and mRNA, and analyzed the enriched pathways. We found that the gene corresponding to these differentially expressed circRNA and lncRNA mainly involved in regulation of the host cell immunity and inflammation, substance and energy metabolism, cell cycle and cell apoptosis. There is also a degree of overlap between the two in these types. In addition, we also found that 114 circRNA and 10 lncRNA with differential changes were related to exosomes. These results suggest that differentially expressed circRNA and lncRNA may be involved in the regulation of these cellular processes. We speculated that the changes in expression of circRNA and lncRNA might provide a new way to explore the pathogenesis of COVID-19.

In addition, in some studies on the clinical manifestations of COVID-19 patients, it has been pointed out that some patients are accompanied by neurological symptoms, including acute cerebrovascular disease symptoms, such as sudden uncontrollable or incoherent talkativeness and limb paralysis; intracranial infection symptoms, such as headache, epilepsy, and disturbance of consciousness; muscle damage symptoms, such as limb soreness and weakness. Neuralgia, paresthesia, defecation and other symptoms^56,57^, as well as the injury of multiple organs such as digestive tract^58,59^, kidney^60^ and lung^61^, have been observed on a small number of patients. In this study, through GO and KEGG enrichment analysis, the differential circRNA and lncRNA are found to play a role in neurons, lung cancer, and many other aspects, in the injury of organs. The study in this paper may, to some extent, help us to understand the causes of these symptoms. Further study from this perspective will help us to further understand why these symptoms appear in COVID-19 patients.

In summary, through detailed bioinformatics analysis of the differential expression of circRNA and lncRNA extracted from the whole blood of recurrent COVID-19 patients, both RNAs, as well as exosome, are confirmed to be relevant, and have the potential to be exploited in understanding, detecting and treating COVID-19. Top 25 circRNAs and lncRNAs in terms of the relevance with COVID-19 are listed in the supplementary file 2, which could be helpful to those research groups that are planning their research in this direction and at the same time have access to COVID-19 samples for qPCR validation.

## Supplementary Information

Supplementary Information 1.

Supplementary Information 2.

## Data Availability

Relevant data has been uploaded to GEO repository, and will be available from Feb 11, 2021. The access number is GSE166552.
