# Supplementary material for: A study of differential circRNA and lncRNA expressions in COVID-19-infected peripheral blood
Source: Sci Rep. 2021 Apr 12;11:7991. doi: 10.1038/s41598-021-86134-0 (PMC8041881; doi:10.1038/s41598-021-86134-0)
Supplement: Supplementary file 1 — Supplementary Information 1. [file 41598_2021_86134_MOESM1_ESM.pdf]

浙江大学医学院附属第四医院

人体研究伦理委员会

临床科研伦理审查批件

批件号 **Approval NO.**: K20200026　　签发日期 **Date of issue**: 2020/2/19

项目名称 **Study Title**	宏基因组二代测序技术在新型冠状病毒临床检测中的应用		
申办方 **Sponsor**	浙江大学医学院附属第四医院		
主要研究者 **Principal Investigator**	夏肖萍	承担专业 **Specialty**	检验科
审查类别 **Category of Review**	初始审查	审查方式 **Type of Review**	快速审查
审查日期 **Date of Review**	2020/2/19	审查地点 **Location of Review**	行政楼 311
审查文件清单 **Reviewed Items**	伦理审查申请表、申报书、知情同意书（版本号：GZ-20200217-01）		
审评意见 **Evaluation**	审查通过。		
审查决定 **Decision**	委员会对该方案的审查决定为: ☑同意（Approval）		
年度/定期跟踪审查 **Continual Review**	1.该研究进行过程中将接受伦理委员会的跟踪审查？☑是(Yes)　□否(No)<br>2.审查频率为该研究批准之日起每　6 月一次，首次请于 2020 年 8 月 19 日前 1 个月递交研究进展报告。<br>3.伦理委员会有根据实际进展情况改变跟踪审查频率的权利。		
（副）主任签字 **Chair Signature**	[signature]		
批准日期 **Approval Date**	2020.2.19		
伦理委员会 **Stamp of ZJEC**	浙江大学医学院附属第四医院人体研究伦理委员会（盖章）		
批件有效期 **Period of Validity**	此批件的有效期为 3 年，逾期未实施的，自行废止。		
