# Supplementary material for: A study of differential circRNA and lncRNA expressions in COVID-19-infected peripheral blood
Source: Sci Rep. 2021 Apr 12;11:7991. doi: 10.1038/s41598-021-86134-0 (PMC8041881; doi:10.1038/s41598-021-86134-0)
Supplement: Supplementary file 2 — Supplementary Information 2. [file 41598_2021_86134_MOESM2_ESM.docx]

Supplementary file 2 for:

A study of differential circRNA and lncRNA expressions in COVID-19-infected peripheral blood

^1#^Yingping Wu, ^2,#^Tiejun Zhao, ^2,*^Riqiang Deng, ^1,*^Xiaoping Xia, ^1^Bin Li, ^2^Xunzhang Wang

^#^: equal contribution

^*^: Correspondence to [lssdrq@mail.sysu.edu.cn, xiaopingxia@zju.edu.cn]

^1^The Fourth Affiliated Hospital Zhejiang University School of Medicine, College of Medicine, Zhejiang University

^2^School of Life Science, Sun Yat-sen University, Guangzhou, 510275, China

**Table 1: Top 25 up-regulated circRNA in the periphery blood of recurrent COVID-19 patients**

| **ID** | ***P* value** | **Fold Change** | **Chromosome** | **Type** | **Gene names** |
| --- | --- | --- | --- | --- | --- |
| hsa_circ_0068443 | 0.006782698 | 6.554371085 | chr3 | exon | DNAJB11 |
| hsa_circ_0031508 | 0.008637436 | 3.733418255 | chr14 | exon | HEATR5A |
| hsa_circ_0017515 | 0.005679824 | 3.00423992 | chr10 | exon | KLF6 |
| hsa_circ_0030333 | 0.007538332 | 2.88923726 | chr13 | exon | TCONS_l2_00007550 |
| hsa_circ_0080221 | 0.000920520 | 2.726414117 | chr7 | exon | EGFR |
| hsa_circ_0089138 | 0.009868183 | 2.543345397 | chr9 | exon | ABL1 |
| hsa_circ_0026309 | 0.000017900 | 2.495213 | chr12 | exon | TFCP2 |
| hsa_circ_0007987 | 0.007613459 | 2.473567608 | chr4 | exon | FAM193A |
| hsa_circ_0044193 | 0.008689216 | 2.417457187 | chr17 | exon | None |
| hsa_circ_0026312 | 0.008918102 | 2.393820376 | chr12 | exon | TFCP2 |
| hsa_circ_0002271 | 0.007076146 | 2.349923394 | chr2 | exon | C2orf43 |
| hsa_circ_0071280 | 0.009033669 | 2.296688837 | chr4 | exon | ARFIP1 |
| hsa_circ_0029996 | 0.006307525 | 2.266183784 | chr13 | exon | DCLK1 |
| hsa_circ_0008864 | 0.007220157 | 2.23434947 | chr4 | exon | SLIT2 |
| hsa_circ_0080135 | 0.006114040 | 2.197338157 | chr7 | exon | TNS3 |
| hsa_circ_0067825 | 0.000767840 | 2.183605521 | chr3 | exon | None |
| hsa_circ_0009985 | 0.005045342 | 2.173995119 | chr1 | exon | VPS13D |
| hsa_circ_0059369 | 0.009891208 | 2.165579006 | chr20 | exon | SLC23A2 |
| hsa_circ_0071279 | 0.008270656 | 2.153859664 | chr4 | exon | ARFIP1 |
| hsa_circ_0054877 | 0.002281165 | 2.15326401 | chr2 | exon | XPO1 |
| hsa_circ_0001314 | 0.006839902 | 2.149427289 | chr3 | exon | FAM208A |
| hsa_circ_0002608 | 0.003514865 | 2.135114203 | chr18 | exon | MIB1 |
| hsa_circ_0005051 | 0.009486015 | 2.079038048 | chr3 | exon | UBXN7 |
| hsa_circ_0054854 | 0.007455790 | 2.069451207 | chr2 | exon | XPO1 |
| hsa_circ_0066226 | 0.004743032 | 2.026226667 | chr3 | exon | FAM208A |

**Table 2: Top 25 down-regulated circRNA in the periphery blood of recurrent COVID-19 patients**

| **ID** | ***P* value** | **Fold Change** | **Chromosome** | **Type** | **Gene names** |
| --- | --- | --- | --- | --- | --- |
| hsa_circ_0053234 | 0.002823665 | 0.202003385 | chr2 | exon | GTF3C2 |
| hsa_circ_0057746 | 0.005733353 | 0.206902389 | chr2 | exon | CFLAR |
| hsa_circ_0064269 | 0.00693875 | 0.230212865 | chr3 | exon | IRAK2 |
| hsa_circ_0011269 | 0.00935953 | 0.244604755 | chr1 | exon | ZCCHC17 |
| hsa_circ_0072269 | 0.002618967 | 0.256282362 | chr5 | exon | NUP155 |
| hsa_circ_0008296 | 0.001791268 | 0.257679737 | chr1 | exon | CTPS |
| hsa_circ_0046692 | 0.001335716 | 0.26472814 | chr18 | exon | YES1 |
| hsa_circ_0052461 | 0.003214859 | 0.286799345 | chr2 | exon | TSSC1 |
| hsa_circ_0051431 | 0.001213956 | 0.288643335 | chr19 | exon | RELB |
| hsa_circ_0003861 | 0.004455151 | 0.292685839 | chr9 | exon | TMEM2 |
| hsa_circ_0087187 | 0.006553844 | 0.296691913 | chr9 | exon | TMEM2 |
| hsa_circ_0047048 | 0.000442178 | 0.310055579 | chr18 | exon | RNMT |
| hsa_circ_0086452 | 0.005026545 | 0.31080634 | chr9 | intron | PLIN2 |
| hsa_circ_0070076 | 0.003496627 | 0.311468142 | chr4 | exon | CNOT6L |
| hsa_circ_0005157 | 0.004096078 | 0.311847856 | chr1 | exon | CTPS |
| hsa_circ_0052731 | 0.003528613 | 0.314065061 | chr2 | exon | NBAS |
| hsa_circ_0075466 | 0.003880633 | 0.315300388 | chr6 | exon | SERPINB9 |
| hsa_circ_0020861 | 0.008160007 | 0.322297841 | chr11 | exon | NUP98 |
| hsa_circ_0071874 | 0.00277159 | 0.328824415 | chr5 | exon | DNAH5 |
| hsa_circ_0075468 | 0.000375461 | 0.333247574 | chr6 | exon | SERPINB9 |
| hsa_circ_0056021 | 0.002933312 | 0.338465084 | chr2 | intron | LIMS3 |
| hsa_circ_0033764 | 0.004781735 | 0.339839729 | chr14 | intergenic  region | ADAM6 |
| hsa_circ_0072771 | 0.004994363 | 0.346162987 | chr5 | exon | CDK7 |
| hsa_circ_0026295 | 0.003199712 | 0.352499903 | chr12 | exon | SLC11A2 |
| hsa_circ_0053234 | 0.002823665 | 0.202003385 | chr2 | exon | GTF3C2 |

**Table 3: Top 25 up-regulated lncRNA in the periphery blood of recurrent COVID-19 patients**

| **ID** | ***P* value** | **Fold Change** | **Chromosome** | **Type** | **Gene Symbol** |
| --- | --- | --- | --- | --- | --- |
| ENST00000504735 | 0.008539915 | 7.881488212 | chr3 | lincRNA | AC016933.1 |
| NONHSAT087070.2 | 0.005514237 | 5.777813993 | chr22 | lncRNA | . |
| NR_037411.1 | 0.00597487 | 5.370602685 | chr20 | precursorRNA | MIR3617 |
| NONHSAT108917.2 | 0.048326389 | 5.069152523 | chr6 | lncRNA | . |
| NONHSAT217169.1 | 0.013444353 | 4.467690568 | chr8 | lncRNA | . |
| ENST00000577988 | 0.020907222 | 4.404294049 | chr17 | snoRNA | SNORD3B-1 |
| lnc-EPAS1-3:5 | 0.044971088 | 4.342351767 | chr2 | lncRNA | . |
| NONHSAT153797.1 | 0.038565592 | 4.286066988 | chr1 | lncRNA | . |
| ENST00000527803 | 0.007930335 | 4.252578129 | chr11 | antisense | AP001922.5 |
| NONHSAT164767.1 | 0.044934461 | 4.191197243 | chr12 | lncRNA | . |
| NONHSAT230460.1 | 0.041869046 | 4.16450549 | chr11 | lncRNA | . |
| ENST00000624260 | 0.035278709 | 4.120926434 | chr17 | lincRNA | AC024610.2 |
| NONHSAT224695.1 | 0.014945753 | 4.119214547 | chr1 | lncRNA | . |
| NONHSAT191457.1 | 0.041718313 | 3.99509951 | chr21 | lncRNA | . |
| ENST00000391111 | 0.010672968 | 3.992838008 | chr10 | snRNA | RNU11-3P |
| NONHSAT222220.1 | 0.018863273 | 3.978251473 | chr9 | lncRNA | . |
| NONHSAT255553.1 | 0.018763253 | 3.961501361 | chr8 | lncRNA | . |
| NONHSAT161665.1 | 0.022137216 | 3.722003116 | chr11 | lncRNA | . |
| NONHSAT192091.1 | 0.016556534 | 3.676256221 | chr22 | lncRNA | . |
| NONHSAT181703.1 | 0.017447923 | 3.591539286 | chr2 | lncRNA | . |
| NR_004407.1 | 0.009833542 | 3.570543464 | chr1 | ncRNA | RNU11 |
| ENST00000591183 | 0.011094501 | 3.553162511 | chr18 | antisense | AC012254.3 |
| NONHSAT162514.1 | 0.006938556 | 3.526318828 | chr12 | lncRNA | . |
| ENST00000387943 | 0.007422584 | 3.518799064 | chr5 | snRNA | RNU6ATAC2P |
| NONHSAT252073.1 | 0.010103726 | 3.510831577 | chr6 | lncRNA | . |

**Table 4: Top 25 down-regulated circRNA in the periphery blood of recurrent COVID-19 patients**

| **ID** | ***P* value** | **Fold Change** | **Chromosome** | **Type** | **Gene Symbol** |
| --- | --- | --- | --- | --- | --- |
| NONHSAT074954.2 | 0.015080461 | 0.063640758 | chr2 | lncRNA | . |
| NR_026880.1 | 0.022732114 | 0.096092806 | chr17 | ncRNA | MGC12916 |
| ENST00000491934 | 0.046805024 | 0.099824206 | chr10 | antisense | DDIT4-AS1 |
| XR_938576.1 | 0.039910996 | 0.141646606 | chrX | ncRNA | LOC105373331 |
| lnc-MAGEB1-2:1 | 0.025176802 | 0.144135294 | chrX | lncRNA | . |
| NONHSAT058415.2 | 0.00232441 | 0.180430441 | chr18 | lncRNA | . |
| XR_001754348.1 | 0.013793427 | 0.181129065 | chr20 | miscRNA | APMAP |
| NONHSAT227543.1 | 0.0198515 | 0.185682666 | chr1 | lncRNA | . |
| NR_027256.1 | 0.046482621 | 0.185814551 | chr6 | misc_RNA | TREML3P |
| NONHSAT128145.2 | 0.00430499 | 0.189499319 | chr8 | lncRNA | . |
| NONHSAT029869.2 | 0.003044504 | 0.190970192 | chr12 | lncRNA | . |
| NONHSAT156320.1 | 0.003608965 | 0.191177538 | chr10 | lncRNA | . |
| NONHSAT106854.2 | 0.002039299 | 0.196948523 | chr6 | lncRNA | . |
| XR_937867.2 | 0.010473426 | 0.19852762 | chr22 | misc_RNA | UPB1 |
| NONHSAT047586.2 | 0.046562091 | 0.198883927 | chr15 | lncRNA | . |
| NONHSAT183568.1 | 0.030962608 | 0.200437875 | chr2 | lncRNA | . |
| ENST00000432032 | 0.015539076 | 0.201942499 | chr22 | antisense | AP000355.1 |
| NONHSAT106828.2 | 0.013086072 | 0.206636316 | chr6 | lncRNA | . |
| NONHSAT107638.2 | 0.007310364 | 0.210369519 | chr6 | lncRNA | . |
| NONHSAT185364.1 | 0.013990622 | 0.215225253 | chr2 | lncRNA | . |
| NONHSAT177410.1 | 0.032282665 | 0.215894222 | chr18 | lncRNA | . |
| NONHSAT018823.2 | 0.013545414 | 0.217210006 | chr11 | lncRNA | . |
| NONHSAT231436.1 | 0.00663306 | 0.219375153 | chr12 | lncRNA | . |
| NONHSAT232053.1 | 0.027304672 | 0.223088933 | chr12 | lncRNA | . |
| NONHSAT122861.2 | 0.028157985 | 0.225890174 | chr7 | lncRNA | . |

**Table 5: Top 25 up-regulated mRNA in the periphery blood of recurrent COVID-19 patients**

| **Gene Symbol** | ***P* value** | **Fold Change** | **Description** |
| --- | --- | --- | --- |
| FOXA2 | 0.009065394 | 3.890792458 | Homo sapiens forkhead box A2 (FOXA2), transcript variant 1, mRNA [NM_021784] |
| RGS7BP | 0.009260059 | 3.580620812 | Homo sapiens regulator of G-protein signaling 7 binding protein (RGS7BP), transcript variant 1, mRNA [NM_001029875] |
| MYO5B | 0.001428247 | 3.137132966 | Homo sapiens myosin VB (MYO5B), mRNA [NM_001080467] |
| LRRC27 | 0.001137017 | 2.926327434 | Homo sapiens leucine rich repeat containing 27 (LRRC27), transcript variant 2, mRNA [NM_001143757] |
| HIST2H2AC | 0.005456767 | 2.892418823 | Homo sapiens histone cluster 2, H2ac (HIST2H2AC), mRNA [NM_003517] |
| SPATA31E1 | 8.74E-05 | 2.873416724 | Homo sapiens SPATA31 subfamily E, member 1 (SPATA31E1), mRNA [NM_178828] |
| TMEM79 | 0.008535874 | 2.867287468 | Homo sapiens transmembrane protein 79 (TMEM79), transcript variant 1, mRNA [NM_032323] |
| HIST2H2BE | 0.00989472 | 2.730128777 | Homo sapiens histone cluster 2, H2be (HIST2H2BE), mRNA [NM_003528] |
| OR5M11 | 0.007978473 | 2.694653976 | Homo sapiens olfactory receptor, family 5, subfamily M, member 11 (OR5M11), mRNA [NM_001005245] |
| TRANK1 | 8.32E-05 | 2.64141816 | Homo sapiens tetratricopeptide repeat and ankyrin repeat containing 1 (TRANK1), mRNA [NM_014831] |
| ST6GALNAC5 | 0.006479574 | 2.639326784 | Homo sapiens ST6 (alpha-N-acetyl-neuraminyl-2,3-beta-galactosyl-1,3)-N-acetylgalactosaminide alpha-2,6-sialyltransferase 5 (ST6GALNAC5), mRNA [NM_030965] |
| ISLR2 | 0.006256333 | 2.565399979 | Homo sapiens immunoglobulin superfamily containing leucine-rich repeat 2 (ISLR2), transcript variant 1, mRNA [NM_001130136] |
| HIST1H2AL | 0.009304502 | 2.502526883 | Homo sapiens histone cluster 1, H2al (HIST1H2AL), mRNA [NM_003511] |
| HAND1 | 0.003905874 | 2.45899257 | Homo sapiens heart and neural crest derivatives expressed 1 (HAND1), mRNA [NM_004821] |
| TMEM221 | 0.002733302 | 2.402948659 | Homo sapiens transmembrane protein 221 (TMEM221), mRNA [NM_001190844] |

**Table 6: Top 25 down-regulated mRNA in the periphery blood of recurrent COVID-19 patients**

| **Gene Symbol** | ***P* value** | **Fold Change** | **Description** |
| --- | --- | --- | --- |
| FFAR3 | 0.00515197 | 0.029389109 | Homo sapiens free fatty acid receptor 3 (FFAR3), mRNA [NM_005304] |
| IL1B | 0.005279832 | 0.044181327 | Homo sapiens interleukin 1, beta (IL1B), mRNA [NM_000576] |
| CD274 | 0.009476039 | 0.057315639 | Homo sapiens CD274 molecule (CD274), transcript variant 1, mRNA [NM_014143] |
| IRAK2 | 0.007421858 | 0.07640952 | Homo sapiens interleukin-1 receptor-associated kinase 2 (IRAK2), mRNA [NM_001570] |
| CSF2 | 0.007786091 | 0.096046798 | Homo sapiens colony stimulating factor 2 (granulocyte-macrophage) (CSF2), mRNA [NM_000758] |
| MS4A12 | 0.005263635 | 0.109792353 | Homo sapiens membrane-spanning 4-domains, subfamily A, member 12 (MS4A12), transcript variant 1, mRNA [NM_017716] |
| CCR9 | 0.003336213 | 0.120966783 | Homo sapiens chemokine (C-C motif) receptor 9 (CCR9), transcript variant C, mRNA [NM_001256369] |
| RNF144B | 0.005234445 | 0.1324896 | Homo sapiens ring finger protein 144B (RNF144B), mRNA [NM_182757] |
| NFKBID | 0.00696554 | 0.133264309 | Homo sapiens nuclear factor of kappa light polypeptide gene enhancer in B-cells inhibitor, delta (NFKBID), mRNA [NM_139239] |
| FZD6 | 0.006950657 | 0.168592412 | Homo sapiens frizzled class receptor 6 (FZD6), transcript variant 1, mRNA [NM_003506] |
| NBN | 0.006840237 | 0.180970046 | Homo sapiens nibrin (NBN), mRNA [NM_002485] |
| CTPS1 | 0.00275289 | 0.18497455 | Homo sapiens CTP synthase 1 (CTPS1), transcript variant 1, mRNA [NM_001905] |
| PEX5L | 0.006212904 | 0.192140211 | Homo sapiens peroxisomal biogenesis factor 5-like (PEX5L), transcript variant 1, mRNA [NM_016559] |
| USP36 | 0.006178658 | 0.209363633 | Homo sapiens ubiquitin specific peptidase 36 (USP36), mRNA [NM_025090] |
| SEC14L2 | 0.003193601 | 0.216057864 | Homo sapiens SEC14-like 2 (S. cerevisiae) (SEC14L2), transcript variant 2, mRNA [NM_033382] |

According to the results of the first 25 differentially expressed genes sequenced by RNA transcriptomics in the peripheral blood of recurrent COVID-19 patients, the noticeably up-regulated circRNAs such as hsa_circ_0017515, hsa_circ_0026309, hsa_circ_0026312, hsa_circ_0029996, and hsa_circ_0008864, and down-regulated circRNAs such as hsa_circ_0051431, hsa_circ_0003861, and hsa_circ_0087187, stem from the antiviral immune regulation of the transcript: KLF6 (1), TFCP2 (2), DCLK1 (3), SLIT2 (4), XPO1 (5), RELB (6), TMEM2 (7). The noticeably up-regulated lncRNAs such as ENST00000577988 and NR_004407.1, and down-regulated lncRNAs such as XR_001754348.1, NR_027256.1, and XR_937867.2, come from maternal genes in the pathways associated with immunological diseases such as acute leukemia, acute liver injury, stroke, and cancer, specifically, SNORD3B-1 (8), RNU11 (9), APMAP (10), TREML3 (11), and UPB1 (12).

1. Bakre A, Wu W, Hiscox J, Spann K, Teng MN, Tripp RA. Human respiratory syncytial virus non-structural protein NS1 modifies miR-24 expression via transforming growth factor-β. J Gen Virol. 2015 Nov;96(11):3179-3191.
2. Farooq QUA, Shaukat Z, Aiman S, Zhou T, Li C. A systems biology-driven approach to construct a comprehensive protein interaction network of influenza A virus with its host. BMC Infect Dis. 2020 Jul 6;20(1):480.
3. Ali N, Allam H, Bader T, May R, Basalingappa KM, Berry WL, Chandrakesan P, Qu D, Weygant N, Bronze MS, Umar S, Janknecht R, Sureban SM, Huycke M, Houchen CW. Fluvastatin interferes with hepatitis C virus replication via microtubule bundling and a doublecortin-like kinase-mediated mechanism. PLoS One. 2013 Nov 19;8(11):e80304.
4. Anand AR, Tirumuru Nagaraja, Ganju RK. A novel role for Slit2/Robo1 axis in modulating HIV-1 replication in T cells. AIDS. 2011 Nov 13;25(17):2105-11.
5. Uddin MH, Zonder JA, Azmi AS. Exportin 1 inhibition as antiviral therapy. Drug Discov Today. 2020 Jun 20;25(10):1775–81.
6. Saha I, Jaiswal H, Mishra R, Nel HJ, Schreuder J, Kaushik M, Singh Chauhan K, Singh Rawat B, Thomas R, Naik S, Kumar H, Tailor P. RelB suppresses type I Interferon signaling in dendritic cells. Cell Immunol. 2020 Mar;349:104043.
7. Jia X, Mo Z, Zhao Q, Bao T, Xu W, Gao Z, Peng L, Zhu X. Transcriptome alterations in HepG2 cells induced by shRNA knockdown and overexpression of TMEM2 gene. Biosci Biotechnol Biochem. 2020 Aug;84(8):1576-1584.
8. Zhao C, Yang S, Lu W, Liu J, Wei Y, Guo H, Zhang Y, Shi J. Increased NFATC4 Correlates With Poor Prognosis of AML Through Recruiting Regulatory T Cells. Front Genet. 2020 Nov 27;11:573124.
9. Gopanenko AV, Malygin AA, Tupikin AE, Laktionov PP, Kabilov MR, Karpova GG. Human ribosomal protein eS1 is engaged in cellular events related to processing and functioning of U11 snRNA. Nucleic Acids Res. 2017 Sep 6;45(15):9121-9137.
10. Plubell DL, Fenton AM, Rosario S, Bergstrom P, Wilmarth PA, Clark WM, Zakai NA, Quinn JF, Minnier J, Alkayed NJ, Fazio S, Pamir N. High-Density Lipoprotein Carries Markers That Track With Recovery From Stroke. Circ Res. 2020 Oct 23;127(10):1274-1287.
11. Allcock RJ, Barrow AD, Forbes S, Beck S, Trowsdale J. The human TREM gene cluster at 6p21.1 encodes both activating and inhibitory single IgV domain receptors and includes NKp44. Eur J Immunol. 2003 Feb;33(2):567-77.
12. Yokoi K, Nakajima Y, Matsuoka H, Shinkai Y, Ishihara T, Maeda Y, Kato T, Katsuno H, Masumori K, Kawada K, Yoshikawa T, Ito T, Kurahashi H. Impact of DPYD, DPYS, and UPB1 gene variations on severe drug-related toxicity in patients with cancer. Cancer Sci. 2020 Sep;111(9):3359-3366.
